# The earliest farmers of northwest China exploited grain-fed pheasants not chickens

**DOI:** 10.1038/s41598-020-59316-5

**Published:** 2020-02-13

**Authors:** Loukas Barton, Brittany Bingham, Krithivasan Sankaranarayanan, Cara Monroe, Ariane Thomas, Brian M. Kemp

**Affiliations:** 1 Cultural Resources Division, DUDEK, San Juan Capistrano, CA 92675 USA; 2 Laboratories of Molecular Anthropology and Microbiome Research, University of Oklahoma, Norman, OK 73019 USA; 3 Department of Microbiology and Plant Biology, University of Oklahoma, Norman, OK 73019 USA; 4 Department of Anthropology, University of Oklahoma, Norman, OK 73019 USA; 5 Department of Anthropology, University of Iowa, Iowa City, IA 52242 USA

**Keywords:** Evolutionary ecology, Archaeology, Evolutionary genetics, Biogeography

## Abstract

Though chickens (*Gallus gallus domesticus*) are globally ubiquitous today, the timing, location, and manner of their domestication is contentious. Until recently, archaeologists placed the origin of the domestic chicken in northern China, perhaps as early as 8,000 years ago. Such evidence however complicates our understanding of *how* the chicken was domesticated because its wild progenitor – the red jungle fowl (*Gallus gallus*) – lives in tropical ecosystems and does not exist in northern China today or in the recent past. Increasingly, multiple lines of evidence suggest that many of the archaeological bird remains underlying this northern origins hypothesis have been misidentified. Here we analyze the mitochondrial DNA of some of the earliest purported chickens from the Dadiwan site in northern China and conclude that they are pheasants (*Phasianus colchicus*). Curiously, stable isotope values from the same birds reveal that their diet was heavy in agricultural products (namely millet), meaning that they lived adjacent to or among some of the earliest farming communities in East Asia. We suggest that the exploitation of these baited birds was an important adaptation for early farmers in China’s arid north, and that management practices like these likely played a role in the domestication of animals – including the chicken – in similar contexts throughout the region.

## Introduction

Northern China is one of the few places where agriculture evolved independently ca. 9,000-7,000 calibrated years before the present (cal BP) and with a suite of plant and animal domesticates unique to the region, namely *Panicum* and *Setaria* millets, pigs, dogs, and a medium-size bird most typically identified as chicken^1,2^. The causes of this agricultural revolution are the subject of much debate^3–5^, as is the direct evidence for it^6^. Among the most contentious of these is the role of the chicken (*Gallus gallus domesticus*) in the agricultural origins of East Asia^7–10^. Indeed, there is no scientific consensus on where, when, or how the domestic chicken evolved, despite the fact that chickens are the most ubiquitous domestic animal on earth today^11^.

Chinese archaeological contexts that contain other evidence for agricultural life, often contain bones of medium-sized birds identified as “chicken,” the oldest of which have been touted as the epicenter of chicken domestication. This is problematic because some of the oldest locations are in the arid regions of northern China where the wild ancestor of the domestic chicken - the tropically adapted “red jungle fowl” (*Gallus gallus*) - does not thrive today^12^. Here we present evidence that the birds exploited by the some of the earliest agricultural peoples of arid northwest China were not chickens at all, but rather pheasants (*Phasianus* spp.), and closely related to the wild pheasants that today thrive in the arid environments of northern Eurasia. Importantly, as with other domestic and commensal animals throughout China and around the world, these birds were dependent upon an environment of resources that exists only in an agricultural ecosystem managed and perpetuated by humans.

## Study Specimens

**Figure 1:**
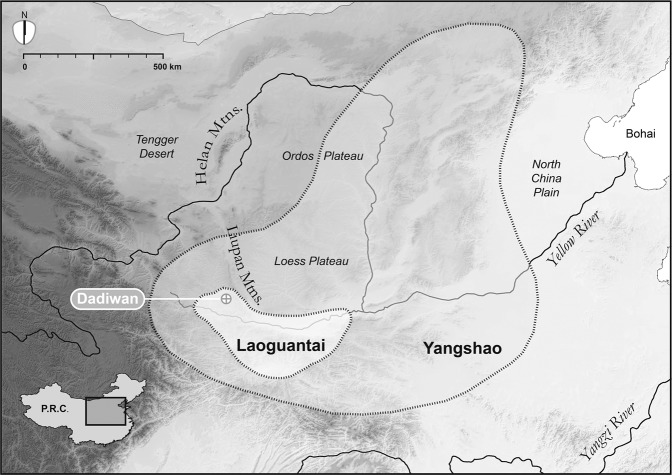
Map of North China showing Dadiwan and the Neolithic culture areas referenced in the text. Regional boundaries are approximate. Basemap is a digital elevation model built from 90 m SRTM data (available at http://srtm.csi.cgiar.org/) using ArcMap 10.7.1 (http://www.esri.com) and Adobe Illustrator CS5 15.0.0 (http://www.adobe.com).

The present study is an extension of a broader effort to understand the origins of agriculture and the co-evolution of domestic plants, animals, and people in arid East Asia^2,3,13,14^. Specimens analyzed here were first collected during the 1978-1984 excavations^15^ of the Dadiwan Neolithic site in Gansu Province, PRC (Fig. 1). Though human hunter-gatherers visited the Dadiwan site sporadically over the past 80,000 years^16^, the earliest evidence for agricultural behavior (i.e., storage features, domestic structures, carbonized remains of cereals, and isotopic evidence that the local dogs had been foddered with those cereals year-round) does not appear until ~7,800 cal BP, during the Laoguantai cultural period^2^. By 6,300 cal BP (the Yangshao cultural period), Neolithic life at Dadiwan was intensively agricultural and culturally complex, with densely packed communities marked by permanent architecture, storage facilities, craft production zones, lavish burials, two kinds of morphologically domestic millets, and isotopic evidence that both dogs, and pigs (in abundance) were provisioned throughout the year with stored grain^2^.

**Figure 2:**
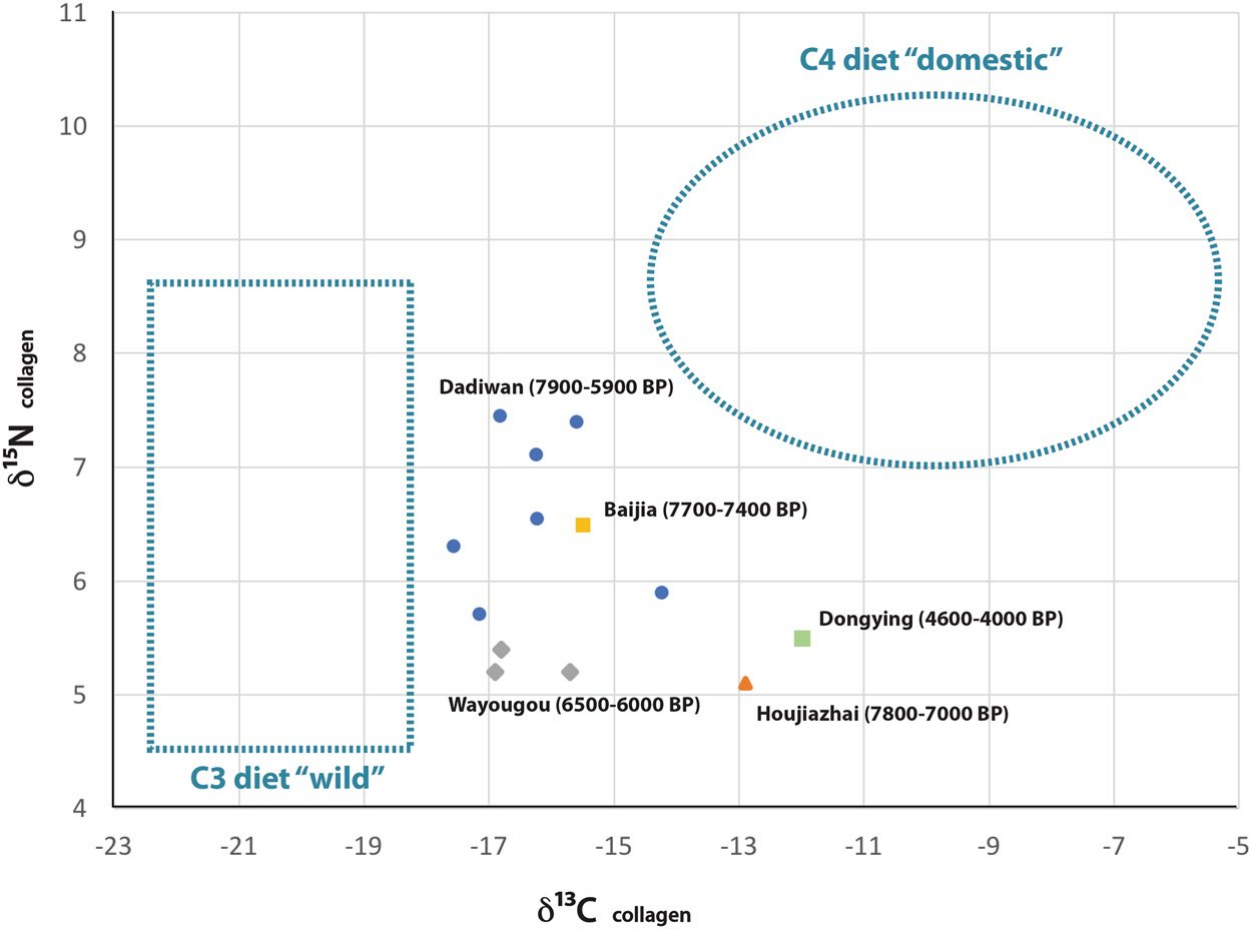
Stable isotope values on bone collagen from Neolithic bird remains from north China established through previously published research^2,40–42^. The “wild” and “domestic” ranges were established from a broad sample of isotopic values from multiple taxa at Dadiwan^2^.

Previous research^2,6^ demonstrates that birds in this Neolithic village farming community were also provisioned with the grains and perhaps vegetation of C_4_ plants (most likely millets), which only grow during the summer. At Dadiwan, the stable isotopic values on the collagen from medium-sized bird bones fall within a range indicative of a year-round diet of both C_4_ and C_3_ vegetation (Fig. 2). This accords well with isotopic differences across the wild-domestic feeding spectrum, for example among modern wild-foraging and pen-raised *Phasianus*^17^, as well as between ancient domestic turkeys (*Meleagris gallopavo*) and contemporary wild turkeys from the American southwest^18^. As with many of the pigs and some of the dogs from Dadiwan, the stable isotope values from the bird bone found at the same site suggest that those birds exploited a mixed-bag of resources, including those they could forage on their own (as if they did not live in the human biome) and those they could only acquire through a life in proximity to human millet cultivators.

Though the Dadiwan birds were initially identified as “*Gallus* sp.,” the original analysts did acknowledge that some of the individuals might also be identified as *Alectoris*, i.e. “chukar” (石鸡) or simply Phasianidae, i.e. “pheasant” (雉)^15^. To resolve the taxonomic uncertainty, we selected for genetic species identification *only* those specimens previously identified as *Gallus* which had already been assayed for stable isotopic proxies of diet. All of these specimens were recovered from buried contexts dating to either the Laoguantai (7,900-7,200 cal BP; n = 3) or Yangshao (6,300-5,900 cal BP; n = 5) cultural periods (Table 1; Supplemental Information Fig. 3). Because the eight specimens come from eight unique depositional contexts and two completely different prehistoric cultural components (separated by ~900 years), initially we assumed them to represent eight different individuals.

**Table 1:** Summary of Dadiwan bird specimens sampled for the current study.

Individual	Provenience	PCR-based ID	NGS-based ID	cal BP	δ^13^C_col_	δ^15^N_col_
LM-088	H398-A181	Phasianus colchicus *	not analyzed	7900-7200	—	—
LM-089	H398-A115	*Phasianus colchicus*	not analyzed	7900-7200	−15.6	7.4
LM-090	F371-A11	*Phasianus colchicus*	not analyzed	7900-7200	−16.8	7.4
LM-091	H393-A93	*Phasianus colchicus*	*Phasianus. colchicus*	6500-5900	−16.2	7.1
LM-092	H227-A140	*Phasianus colchicus*	*Phasianus. colchicus*	6500-5900	−17.6	6.3
LM-093	H227-A52	*Phasianus colchicus*	*Phasianus. colchicus*	6500-5900	−14.2	5.9
LM-094	H227-A50	*Phasianus colchicus*	not enough DNA	6500-5900	−17.2	5.7
LM-095	H227-A53	*Phasianus colchicus*	not analyzed	6500-5900	−16.2	6.5

All specimens were recovered during previous excavations^15^; all chronological assignments^2,15,16^ and all stable isotope data^2^ have been established through previous research. Note: LM-088 did not produce sufficient collagen for isotopic analysis, and despite repeated treatments, we were unable to replicate the initial molecular identification of LM-088. PCR stands for pre-polymerase chain reaction; NGS stands for next generation sequencing.

## Methods

Here we report the methods used for genetic species identification. The methods and rationale behind morphological species identification of zooarchaeological specimens^12,15^, stable isotope analyses of bone collagen^2,6^, and radiocarbon dating of associated materials^2,16^, have all been published elsewhere, and are not described herein. We merely report and evaluate the results of those analyses (Table 1; Fig. 2).

All DNA extraction and pre-polymerase chain reaction (PCR) procedures were conducted in the ancient DNA cleanroom at the Laboratories of Molecular Anthropology and Microbiome Research (LMAMR; lmamr.org) at the University of Oklahoma, Norman, OK. DNA was extracted using ancient DNA methods and various primers were designed to screen the mitochondrial DNA for sections that are informative for discriminating species (Supplemental Information). Once the specimens had been identified as the common ring-necked pheasant (*Phasianus colchicus*), additional primers were designed to sequence the bulk of the control region. Shotgun DNA libraries were constructed from four of the samples – a subset of the total eight – as an additional means of authenticating our findings (Supplemental Information).

All sequences were aligned to the full *Phasianus colchicus* mitochondrial genome (NC_015526.1) in Sequencher (version 5.4.6). A comparative data set of 104 control region mtDNA (spanning from np 170 to 950) from three subspecies of ring-necked pheasant (*P.colchicus alashanicus*, *P.colchicus pallasi*, *P.colchicus strauchi*^19–23^ was compiled from Genbank. These data along with control region sequences from samples LM-091, LM-092, LM-093, and LM-094 were aligned with DNA Alignment (version 1.3.3.2), converted to a Nexus file, and imported into PopART version 1.7^24^. A median-joining network was created using the default parameters in PopART.

These same sequences were aligned using MUSCLE^25^ in Seqotron^26^ prior to manual curation. The Bayesian Information Criterion (BIC)^27^ in jModelTest2^28^ was used to estimate the model of evolution for the control region sequence in *Phasianus colchicus*. A neighbor-joining tree was constructed using PAUP* version 4.0a 165^29^ with HKY + G_I as the model of evolution^30^ at default parameters for an initial evaluation of population structure. A comparative control region sequence from Elliot’s pheasant (*Syrmaticus ellioti*; Genbank accession AB164624) was defined as the outgroup^31^. Following the neighbor-joining tree, a Bayesian analysis sans the outgroup was performed in BEAST 2.5^32^ using the same model of evolution under a constant population prior. Three independent runs sampling every 5,000^th^ generation out of 500 million iterations of the Markov chain Monte Carlo were run. The tip dates of the samples were not included in any of the runs. Each run was then resampled using LogCombiner version 1.10.4 to pull every 50,000^th^ generation. All three runs were combined, and 10% of the burnin removed. The final tree was generated based on maximum clade credibility derived from median node heights.

To confirm the Bayesian analyses, a maximum likelihood using PAUP* was run using the same model, HKY + G + I, with 1000 bootstrap replicates. No bootstrap values exceeded 50% for all internal and terminal nodes. Low bootstrap support in the consensus trees may have been related to the high number of shared haplotypes resulting in distortion. A secondary maximum likelihood was run for the unique haplotypes (n = 54) using the same parameters, and the topology was consistent with both the median-joining network and the Bayesian tree.

Consensus sequences produced from high throughput sequencing were aligned with 34 complete mitochondrial genomes belonging to organisms in the *Phasianinae* sub-family, followed by construction of a Maximum likelihood tree using RAxML^33^ (GTRGAMMA substitution model, 100 bootstrap replicates).

## Results

All specimens analyzed were identified as the common ring-necked pheasant (*Phasianus colchicus*) based on sequences produced with the numerous primer pairs employed in our study (Supplemental Information). Independent amplifications confirm the species identification of seven specimens. Only a single amplicon from sample LM-088 was sequenced successfully using primer set LophuraF/R and identified as the common ring-necked pheasant, however this result could not be replicated. This may be due to the fact that the LM-088 specimen was burned and/or partially calcined in prehistory and contains little intact, original collagen (Table 1, Supplemental Fig. 1). Contamination was not detected in the extraction negative controls or in any PCR negative controls.

**Figure 3:**
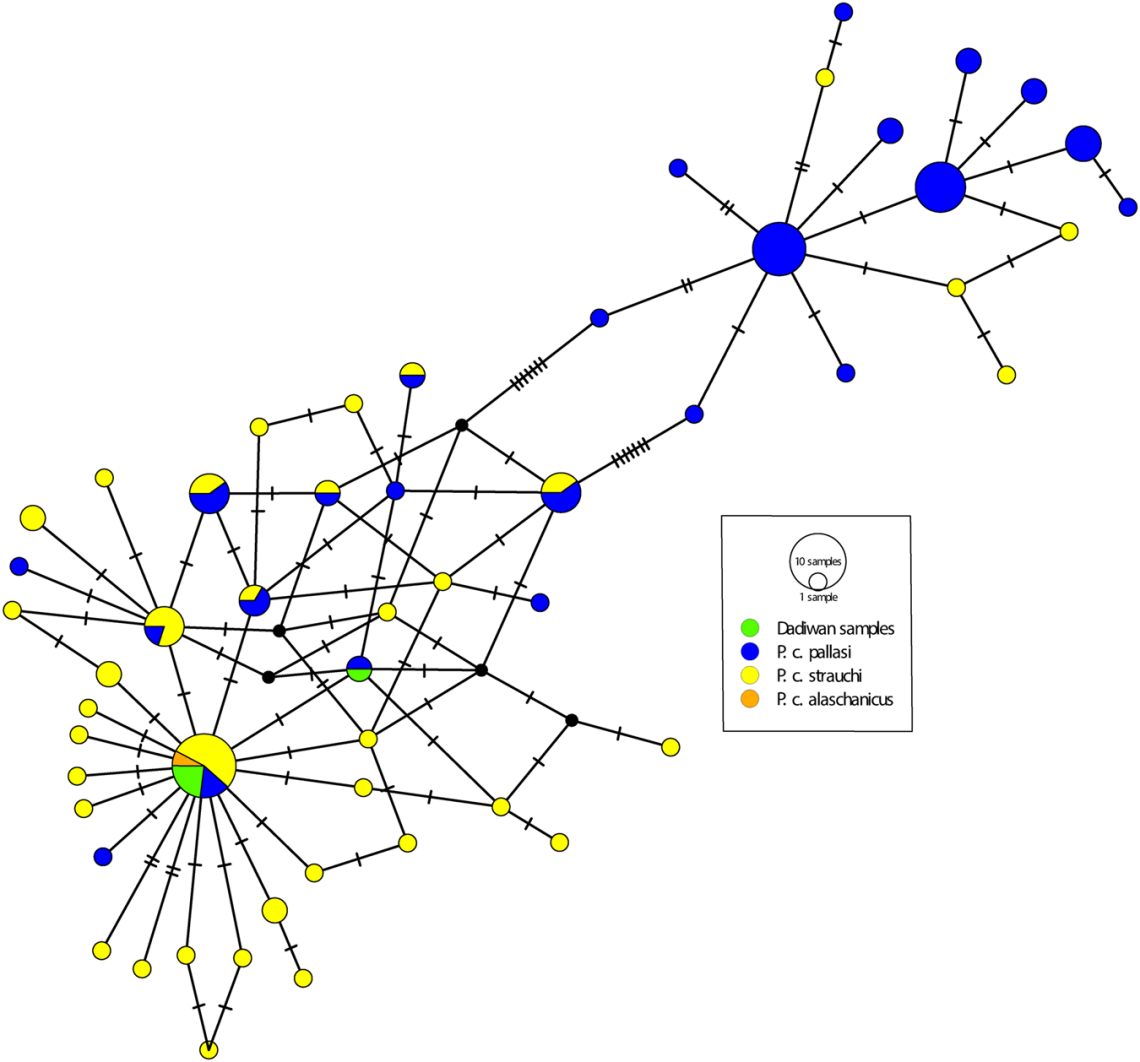
Median-joining network (constructed using 780 bp of the control region that spans from np 170-950) illustrating the relationship between the Dadiwan specimens and 108 comparative control region sequences from Genbank. There are two lineages depicted in the network. One lineage is found in the central clade and is shared by all of the subspecies included. The other lineage is a derived form of the central clade that is shared only with *P. c. pallasi*.

Six sets of control region primers (C149F/C325R, C266F/C438R, C400F/C580R, C539F/C711R, C660F/C833R, and C791F/C971R), were sequenced to further identify at the sub-species level. Four samples (LM-091, LM-092, LM-093, and LM-094) belong to two matrilines, which are shared to published Genbank sequences of three ring-necked pheasant subspecies (*P. c. alaschanicus, P. c. pallasi*, or *P. c. strauchi*). The median-joining network (Fig. 3) and Bayesian tree (Supplemental Fig. 2) illustrate haplotype sharing, and further depict the relationship between the Dadiwan specimens and 108 comparative ring-necked pheasant sequences. Samples LM-089, LM-090, and LM-095 were excluded from this analysis due to lack of comparative mitochondrial control region sequences (i.e., spanning from np 170 to 950). As illustrated in the network, one lineage is found in the central clade and is shared by all subspecies. The other lineage is a derived form of the central clade that is shared only with *P. c. pallasi*. Furthermore, though four of these birds are a 100% match with modern individuals belonging to three regional ring-necked pheasant subspecies: “strauchi”, “pallasi” and “alashanicus” (Fig. 3), they are genetically distinct from the dozens of other subspecies found elsewhere throughout Eurasia. The Bayesian tree is congruent with the network, illustrating two clades with strong support (Supplemental Fig. 2). Both internal and terminal nodes are poorly supported with posterior probabilities thus, the trees are not accurate representations of evolutionary divergence. More genetic information is required to full resolve the phylogenetic placement of both modern and ancient *Phasianus colchicus* subspecies. The topologies reveal a general pattern that suggests the hypervariable region of the *Phasianus* mitochondrial lineages are very similar across subspecies with a likelihood of lineage sharing. More genetic information is required to fully resolve the phylogenetic placement of *Phasianus colchicus* subspecies in this analysis. This would involve generating full mitogenome sequences from both ancient and contemporary specimens to supplement the few *Phasianus* mitogenomes currently available in genomic databases.

**Figure 4:**
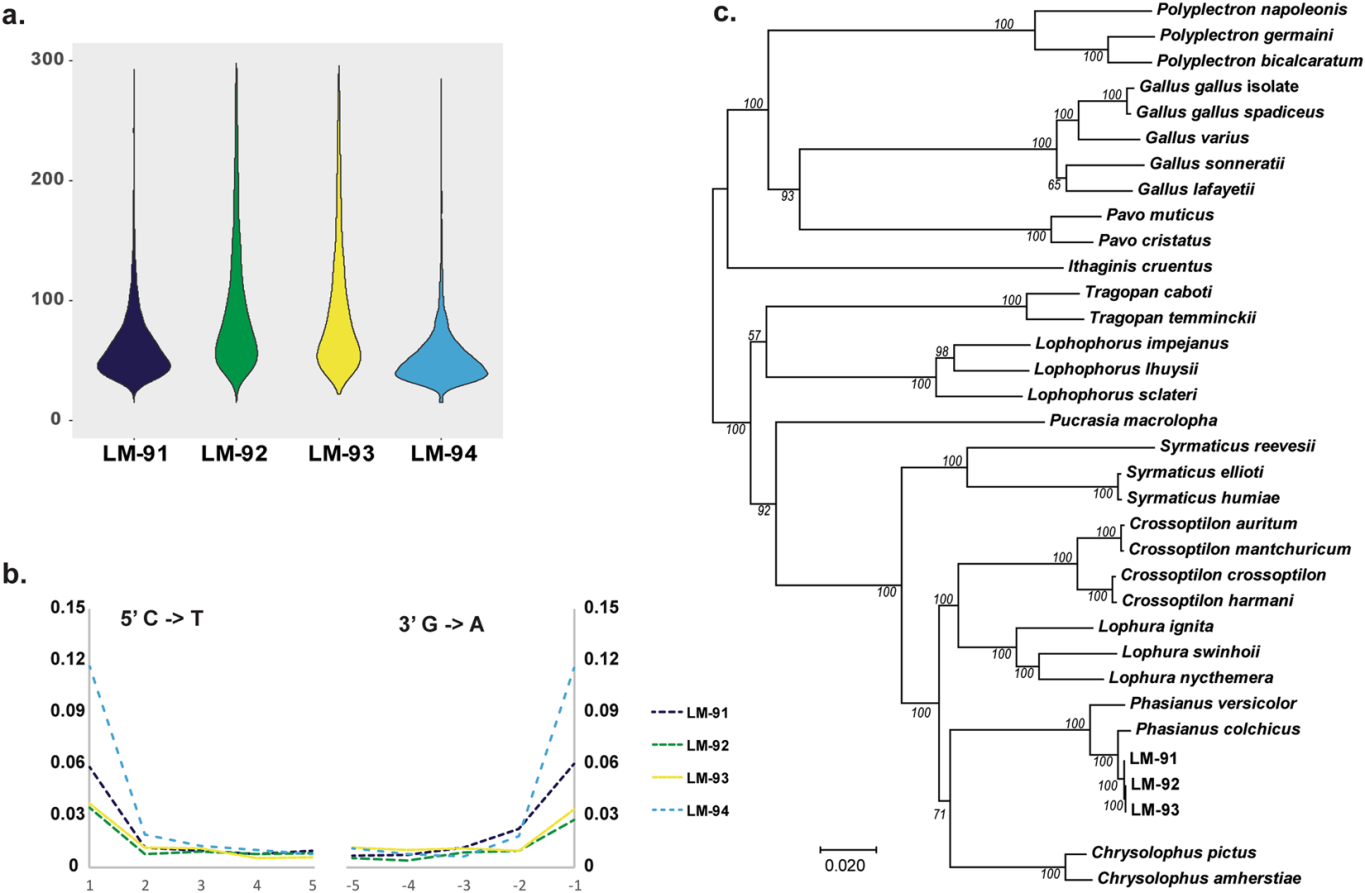
Authentication of endogenous ancient DNA. (**a**) Fragment size distribution of reads mapped to *P. colchicus* genome, (**b**) Terminal base damage rates for reads mapped to *P. colchicus* genome, and (**c**) Maximum likelihood tree built from whole mitochondrial genomes depicting phylogenetic placement of Dadiwan samples in relation to members of the sub-family *Phasianinae*. Sample LM-094 was removed prior to phylogenetic analysis due to limited sequence coverage (127 sites with >5x coverage).

A total of 18.4 million read pairs were generated across the four sample libraries (4.7 M ± 1.8 M; mean ± s.d.). Post-quality filtering, between 80–91% of the reads were retained for samples LM-091, LM-092, and LM-093 (Supplemental Table 4). Comparison of analysis-ready reads from these three samples to the *P. colchicus* reference genome (GCA_004143745) resulted in mapping rates of between 79–90%. Sample LM-094 had a large fraction of adapter dimers (~56%), attributed to the low starting DNA quantities used during library preparation. Consequently, read mapping rates for LM-094 when compared to the *P. colchicus* reference genome was ~9% (Supplemental Table 4). Fragment distributions for mapped reads are consistent with those expected for ancient DNA specimens^34,35^, ranging between a median fragment size of 47–76 bp for the four libraries (Fig. 4a, Supplemental Table 4). Terminal deamination follows patterns expected for ancient DNA libraries generated with partial-UDG treatment^36,37^, with 5′ C- > T rates between 3.4%–11.7%, and 3′ G- > A rates between 2.8%–11.6% (Fig. 4b, Supplemental Table 4). Mapping of analysis-ready reads from samples LM-091, LM-092, and LM-093, to the *P. colchicus* mitochondrial genome (NC_015526) showed coverage with five reads or higher across >99% of sites along the reference sequence. Phylogenetic analysis of consensus mtDNA sequences generated from these alignments showed clustering of these specimens with *P. colchicus* (100% bootstrap support; Fig. 4c).

Based on the similarities and differences of age, archaeological provenience, skeletal element morphology, diet, and mitogenome, the assemblage of bird bone presented here could come from as few as four but as many as eight different individual birds (Supplemental Information, Supplemental Tables 6 and 7).

## Discussion

Because Dadiwan is among the oldest unequivocally agricultural settlements in China, and the earliest in the arid northwest, a fuller understanding of agricultural evolution, and potentially chicken domestication, requires greater confidence in the taxonomic identity of the faunal remains recovered at the site.

Our analyses reveal that the birds living in the human biome surrounding the Dadiwan site, and subsequently exploited by people, were neither domestic chickens, nor their wild ancestors the red jungle fowl. Instead, we identify them as pheasants (*Phasianus colchicus*). Importantly, the three extant subspecies that match the prehistoric individuals from Dadiwan are all found in northern China today in a mixture of northern latitude environments that include deserts, steppe, and arid highlands^38^. Moreover, when the Dadiwan sequences were compared to the tropical taxa of Asia they deviated greatly, suggesting that the prehistoric provisioning and exploitation of large meaty birds at Dadiwan was a local (i.e. not imported) adaptation.

Currently we have no evidence that these Dadiwan birds were kept in pens, that they provided eggs for human consumption, or even that they were under direct human control. What we can say is they do not resemble wild-foraging animals, and that they must have spent a sizeable portion of their lives eating things that only humans could provide. In turn, the humans ate the birds fed either on their own grain stores, or on the vegetative waste associated with the harvesting and processing of these domesticated millets.

We refer to this simple symbiosis as “low-level bird production” and suggest that it was an intentional, and low-cost human strategy for enhancing local resource yields. Because the abundance of birds could be tuned up or down simply by modifying the abundance of agricultural waste (either within the village grounds, or in the adjacent agricultural plots), it was an effective means of low-level food production^39^. Furthermore, the practice could be initiated every time the farming community fissioned or relocated, without having to transport caged or incubated birds. Birds endemic to the local environmental context simply preyed upon the village refuse in pursuit of their own self-interest and in turn the people preyed upon those birds.

The isotopic pattern in bird bone collagen is repeated elsewhere in Neolithic north China, suggesting the practice of low-level bird production was adaptive across a broad swath of ethno-linguistic groups inhabiting temperate environmental contexts, through time and space (Figs. 1 and 2). A spatio-temporal study of archaeological bird remains like these might reveal whether low-level bird production was limited to pheasants, and may provide insight about the degree to which pheasants, or any other large, meaty, ground-dwelling birds – including chickens and their wild ancestors – might have been managed in prehistory.

## Supplementary information

Supplementary Information.

## Data Availability

All data generated or analyzed during this study are included in this published article and its Supplementary Information files. Complete sequence data have been submitted to NCBI under the accession ID PRJNA578639 (https://www.ncbi.nlm.nih.gov/bioproject/PRJNA578639).
