# Supplementary material for: The earliest farmers of northwest China exploited grain-fed pheasants not chickens
Source: Sci Rep. 2020 Feb 13;10:2556. doi: 10.1038/s41598-020-59316-5 (PMC7018827; doi:10.1038/s41598-020-59316-5)
Supplement: Supplementary file 1 — Supplementary Information. [file 41598_2020_59316_MOESM1_ESM.pdf]

Supplemental Information

The earliest farmers of northwest China exploited grain-fed pheasants not chickens.

Loukas BARTON[1], Brittany BINGHAM[2],* Krithivasan SANKARANARAYANAN[2,3], *Cara MONROE[2,4], Ariane THOMAS[5], Brian M. KEMP[2,4]*

1. Cultural Resources Division, DUDEK, San Juan Capistrano, CA 92675 USA
2. Laboratories of Molecular Anthropology and Microbiome Research, University of Oklahoma, Norman, OK 73019 USA
3. Department of Microbiology and Plant Biology, University of Oklahoma, Norman, OK 73019 USA
4. Department of Anthropology, University of Oklahoma, Norman, OK 73019 USA
5. Department of Anthropology, University of Iowa, Iowa City, IA 52242 USA

Methods and Results

DNA Extraction

All DNA extraction and pre-polymerase chain reaction (PCR) procedures were conducted in the ancient DNA cleanroom at the Laboratories of Molecular Anthropology and Microbiome Research (LMAMR; lmamr.org) at the University of Oklahoma, Norman, Oklahoma. This facility is a dedicated workspace for processing aged, degraded, and/or low copy number DNA samples. Precautions aimed to minimize and monitor the introduction of contamination are practiced in the laboratory.

DNA was extracted from the specimens following Kemp et al. [1]. Approximately 50 mg or less of bone material was subsampled from each specimen. These subsamples were submerged in 6% (w/v) sodium hypochlorite for 4 min [2]. The sodium hypochlorite was poured off and the samples submerged in DNA-free water, which was immediately poured off. The samples were once again submerged in DNA-free water and the water poured off.

The bone samples were transferred to 1.5 mL tubes, to which aliquots of 500 µL of ethylenediaminetetraacetic acid (EDTA) were added, and the tubes gently rocked at room temperature for >48 hours. An extraction negative control, to which no bone material was added, accompanied each batch of extractions to monitor for possible contamination.

Ninety µL of proteinase K (BIOBASIC cat # 32181) at a concentration of 1 mg/30 µL (or >20 Units/30 µL) was added to each sample, and the tubes incubated at 64-65°C for 3 hours. Following proteinase K digestion, the tubes were centrifuged at 14,000 revolutions per minute (rpm) for one minute to pellet any undigested bone, dirt, and/or “sludge”. All centrifugation steps in this study were conducted with an Eppenddorf centrifuge 5424. The liquid was carefully moved to a new 1.5 mL tube, to which 750 µL

of 2.5% “Resin” (i.e., 2.5% celite in 6M guanidine HCl) and 250 µL of 6M guanidine HCl were be added. The tubes were vortexed multiple times over approximately a 2 min period.

Promega Wizard minicolumns were attached to 3 mL luer-lok syringe barrels (minus the plunger) and placed on a vacuum manifold. Three mL of DNA-free water was first pulled across the columns with the intent to wash away potential contaminating DNA. The DNA/Resin mixture was subsequently pulled across the columns. The silica pelleted on the minicolumns was rinsed by pulling 3 mL of 80% isopropanol across the columns.

The minicolumns were then placed in new 1.5 mL tubes and centrifuged at 10,000 rpm for 2 minutes to remove excess isopropanol. The minicolumns were transferred to new 1.5 mL tubes. Fifty µL of DNA-free water heated to 64-65°C was added to the minicolumns and left for 3 min before centrifugation of the tubes for 30 seconds at 10,000 rpm. This step was repeated, amounting to 100 µL of extracted DNA.

Extractions were tested for inhibition following [1], using DNA extracted from archaeological turkey specimens [3]. Two samples were prevented from amplifying turkey control DNA (LM-090 and LM-093); the samples were subjected to repeat silica extraction (following as describe above). After the second silica extraction, a subsequent inhibition test showed no evidence of inhibition.

<u>Polymerase Chain Reaction</u>

Polymerase chain reactions (PCRs) were then performed following [1]. PCR reactions contained 1X Omni Klentaq Reaction Buffer (including a final concentration of 3.5 mM $MgCl_2$), 0.32 mM dNTPs, 0.24 µM of each primer, 0.3 U of Omni Klentaq LA polymerase, and 1.5 µL of template DNA. Following denaturing at 94°C for 3 minutes, 60 cycles of PCR was conducted at 94°C for 15 s, the annealing temperature for 15 s (Supplemental Table 1), and 68°C (note that this is the optimal extension temperature for Omni Klentaq LA polymerase) for 15 s. PCR negative controls were used to monitor for possible contamination.

First, cytochrome oxidase I (COI) gene primers (COI-F/R; Supplemental Table 1) described by Xiang et al. [4] were used to determine if the samples were *Gallus* spp. or *Phasianus* spp. According to Xiang et al. [4], “The COI primers were degenerate by design to allow amplification of both *Gallus* (NC_001323) and *Phasianus* (NC_015526) sequences.” However, we were to later to learn that, in fact, these primers bias against *Phasianus* (as assessed in the program Amplify4). This might indicate that the negative results reported by Xiang et al. [4] are the product of the remains they studied being non-*Gallus*. The possible extent of bias against birds outside of the genus *Gallus* is unknown. Nevertheless, these primers were designed to produce 156 base pairs (bp) amplicons spanning from nucleotide position (np) 7038 to 7193 relative to the domestic chicken (*Gallus gallus*) full mitochondrial DNA reference sequence (Genbank accession

NC_001323.1). No DNA was amplified using these primers, indicating that the samples were either not *Gallus* or that there was no amplifiable DNA present in the samples.

Next, we employed a primer set (AmbigF1/R1; Supplemental Table 1) developed by our research group to identify North American birds in general, based on the COI sequences of over 600 species [5]. While they were designed to work on North American species, we thought they might be useful in discriminating Asian species as well. Three of the samples (LM-090, LM-091, and LM-094) amplified using these primers, and the sequences identified the samples as *Phasianus colchicus*, or the common ring-necked pheasant.

In order to possibly improve amplification, PCRs were also performed using both PCR Enhancer Cocktail P (PEC-P; DNA Polymerase Technology; see also Zhang et al. [6] 2and Rescue PCR. PEC-P PCRs were performed at 20% v/v as described by Palmer et al. [7] . Each reaction contained the following: 1X Omni Klentaq Reaction Buffer (including a final concentration of 3.5 mM $MgCl_2$), 0.32 mM dNTPs, 0.24 µM of each primer, 0.3 U of Omni Klentaq LA polymerase, 20% (v/v) PCR enhancer cocktail, and 1.5 µL of template DNA. Rescue PCR is a reagent-rich PCR mix and was performed at 25% following Johnson and Kemp (2017). Each rescue PCR contained: 1.25X Omni Klentaq Reaction Buffer (including a final concentration of 4.375 mM $MgCl_2$), 0.4 mM dNTPs, 0.3 µM of each primer, 0.375 U of Omni Klentaq LA polymerase, and 1.5 µL of template DNA. In both cases, PCR negative controls were used to monitor for possible contamination.

After determining that three samples were *P. colchicus*, nine primer sets were designed that would allow us to discriminate between 20 species of medium sized birds found in northern China and parts of SE Asia (Supplemental Table 1). The first primer set (GallusF/R) was designed to amplify a 167 bp region of the COI gene, spanning nucleotide position np 7132 to 7298 of the domestic chicken (*Gallus gallus*) full mitochondrial DNA reference sequence (Genbank accession KX987152.1). Sequences of this amplicon spanning np 7153 to 7279 can be used as a barcode to differentiate between the domestic chicken (*Gallus gallus*) and the Grey Junglefowl (*Gallus sonneratii*).

The second primer (SyrmF/R) was designed to amplify a 196 bp region of the 12S gene, spanning nucleotide position np 1492 to 1687 of the Reeves's pheasant (*Syrmaticus reevesii*) full mitochondrial DNA reference sequence (Genbank accession NC_010770.1). Sequences of this amplicon spanning np 1512 to 1666 can be used as a barcode to discriminate between the Reeves's pheasant, Elliot's pheasant (*Syrmaticus ellioti*), and Mrs. Hume's pheasant (*Syrmaticus humiae*).

The third primer set (LophuraF/R) was designed to amplify a 189 bp region of the COI gene, spanning nucleotide position 70 to 258 of the Silver pheasant (*Lophura nycthemera*) full COI gene reference sequence (Genbank accession JN709934.1).

Sequences of this amplicon spanning 91 to 239 can be used as a barcode to discriminate between the Silver pheasant and the Edwards’s pheasant (*Lophura edwardsii*).

The fourth primer set (TetraF/R) was designed to amplify a 198 bp region of the COI gene, spanning nucleotide position 6661 to 6858 of the Verreaux’s monal-partridge (*Tetraophasis obscurus*) full mitochondrial DNA reference sequence (Genbank accession JF921876.1). Sequences of this amplicon spanning 6679 to 6839 can be used as a barcode to discriminate between the Verreaux’s monal-partridge and the Szechenyi's monal-partridge (*Tetraophasis szechenyii*).

The fifth primer set (Pheas1F/R) was designed to amplify a 198 bp region of the COI gene, spanning nucleotide position 6821 to 7018 of the Brown eared phesant (*Crossoptilon mantchuricum*) full mitochondrial DNA reference sequence (Genbank accession KY070317.1). Sequences of this amplicon spanning 6840 to 6999 can be used as a barcode to discriminate between the Brown eared pheasant and the Blood pheasant (*Ithaginis cruentus*).

The sixth primer set (Pheas2F/R) was designed to amplify a 162 bp region of the COI gene, spanning nucleotide position 6723 to 6884 of the common ring-necked pheasant (*Phasianus colchicus*) full mitochondrial DNA reference sequence (Genbank accession NC_015526.1). Sequences of this amplicon spanning 6743 to 6866 can be used as a barcode to discriminate between the common ring-necked pheasant, the Koklass pheasant (*Pucrasia macrolopha*), and the Golden pheasant (*Chrysolophus pictus*).

The seventh primer set (Pheas3F/R) was designed to amplify a 195 bp region of the COI gene, spanning nucleotide position 325 to 519 of the Altai snowcock (*Tetraogallus altaicus*) full COI gene reference sequence (Genbank accession GQ482760.1). Sequences of this amplicon spanning 344 to 500 can be used as a barcode to discriminate between the Altai snowcock and the Temminck's tragopan (*Tragopan temminckii*).

The eight primer set (PartF/R) was designed to amplify a 204 bp region of the Cytochrome b (cytb) gene, spanning nucleotide position 62 to 265 of the Przevalski's partridge (*Alectoris magna*) full cytb region reference sequence (Genbank accession HQ727950.1). Sequences of this amplicon spanning 80 to 245 can be used as a barcode to discriminate between the Przevalski’s partridge, the Chukar (*Alectoris chukar*), and the Daurian partridge (*Perdix dauurica*).

The final primer set (OtisF/R) was designed to amplify a 186 bp region of the COI gene, spanning nucleotide position 63 to 248 of the Great Bustard (*Otis tarda*) full COI gene reference sequence (Genbank accession KY754526.1). Sequences of this amplicon spanning 82 to 227 can be used as a barcode to discriminate between the Great Bustard and the Little Bustard (*Tetrax tetrax*).

A new, primer set (Pheas6841F/Pheas7015R) was designed to substitute for AmbigF1/R1, making them specific to the common ring-necked pheasant (*Phasianus colchicus*) (Supplemental Table 1). These primers amplify a 175 bp region of the COI gene, spanning nucleotide position 6841 to 7015 of the common ring-necked pheasant full mitochondrial DNA reference sequence (Genbank accession NC_015526.1). Sequences of this amplicon spanning 6863 to 6993 can be used as a barcode for the common ring-necked pheasant.

Another primer set (C149F/C325R) was created to sequence a 177 base-pair stretch of the mitochondrial control region, spanning nucleotide position 149 to 325 of the common ring-necked pheasant full mitochondrial DNA reference sequence (Genbank accession NC_015526.1). Given sufficient amplification across 6 out of the 8 samples using the C149F/C325R, seven additional primer sets (Supplemental Table 1) were designed to amplify short (≤186 bp), overlapping sections of the control region in an attempt to possibly narrow down our identifications to the subspecies level. Control region sequences were also used to build a median-joining network and phylogenetic tree, as described below.

Following the above described methods, samples LM-088, LM-089, LM-090, LM-091, LM-094, and LM-095 were identified as the common ring-necked pheasant and portions of their mitochondrial DNA control region were sequenced (except LM-088 which failed to produce control region amplicons).

<u>Second Round of DNA Extraction</u>

Two samples (LM-092 and LM-093) failed to produce amplicons following the methods described above and one sample (LM-088) had only produced one amplicon. In this case we again subsampled ≤50 mg from these specimens for an alternative DNA extraction method. These subsamples were submerged in 6% (w/v) sodium hypochlorite for 4 min [2]. The sodium hypochlorite was poured off and the samples submerged in DNA-free water, which was immediately poured off. The samples were once again submerged in DNA-free water and the water poured off.

The bone samples were transferred to 1.5 mL tubes, to which aliquots of 500 µL of ethylenediaminetetraacetic acid (EDTA) were added, and the tubes gently rocked at room temperature for >48 hours. An extraction negative control, to which no bone material was added, accompanied each batch of extractions to monitor for possible contamination. Ninety µL of proteinase K (BIOBASIC cat # 32181) at a concentration of 1 mg/30 µL (or >20 Units/30 µL) was added to each sample, and the tubes incubated at 64-65°C for 3 hours.

To each tube was added 500 µL of phenol:chloroform:isoamyl alcohol (25:24:1) and the tubes gently rocked for 5 min. The tubes were centrifuged at 14,000 rpm for 5 min and the aqueous phases transferred to new tubes. Extraction with

phenol:chloroform:isoamyl alcohol (25:24:1) was repeated and the aqueous phases transferred to new tubes. Then 500 µL of chloroform:isoamyl alcohol (24:1) was added to the tubes, which were rocked gently for 5 min and then centrifuged at 14,000 rpm for 3 min. The aqueous phases were again transferred to new tubes.

The aqueous phases were again transferred to new 1.5 mL tubes, to which 750 µL of 2.5% "Resin" (i.e., 2.5% celite in 6M guanidine HCl) and 250 µL of 6M guanidine HCl were be added. The tubes were vortexed multiple times over approximately a 2 min period.

Promega Wizard minicolumns were attached to 3 mL luer-lok syringe barrels (minus the plunger) and placed on a vacuum manifold. Three mL of DNA-free water was first pulled across the columns with the intent to wash away potential contaminating DNA. The DNA/Resin mixture was subsequently pulled across the columns. The silica pelleted on the minicolumns was rinsed by pulling 3 mL of 80% isopropanol across the columns.

The minicolumns were then placed in new 1.5 mL tubes and centrifuged at 10,000 rpm for 2 minutes to remove excess isopropanol. The minicolumns were transferred to new 1.5 mL tubes. Fifty µL of DNA-free water heated to 64-65°C was added to the minicolumns and left for 3 min before centrifugation of the tubes for 30 seconds at 10,000 rpm. This step was repeated, amounting to 100 µL of extracted DNA.

<u>Polymerase Chain Reaction of Samples Extracted a Second Time</u>

The DNA extractions of these three samples (LM-088, LM-092, and LM-093) were tested for inhibition, and treated accordingly, as described above. PCRs were attempted on these three extractions using the barrage of primers describe above and also in supplemental table 1.

From this second round of extraction and PCR amplification, samples LM-092 and LM-093 were identified as *Phasianus colchicus* and portions of their control region were sequenced. Sample LM-088 failed to produce any amplicons.

<u>Authentication of Ancient DNA</u>

Shotgun libraries were constructed with DNA extracts from a subset of the samples (LM-091, LM-092, LM-093 and LM-094). Using 10 µl of DNA extracts, blunt ended-dual indexed Illumina libraries for Next Generation shotgun sequencing were prepared for the four samples, their corresponding extraction control, and a library negative control following the BEST (Blunt End Single Tube) method [8] with some modifications. This included a partial uracil–DNA–glycosylase treatment (to repair some post-mortem damage) prior to end repair [9]. Amplified libraries were purified using 1.5x Sera-Mag magnetic beads, and eluted in 30 µl of EB. The concentration and size distribution of the libraries were estimated with an Agilent Fragment Analyzer and through qPCR using

Kapa Biosystems SYBR® FAST qPCR Master Mix. Dual-indexed shotgun libraries were pooled in equimolar concentrations and sequenced on an Illumina NextSeq (2x150bp) at the Oklahoma Medical Research Foundation, Clinical Genomics Center. Demultiplexed reads were processed using AdapterRemoval (V2)[10] to remove reads with uncalled bases ('N'), filter low quality bases (Q <30), remove Illumina adapter sequences, and merge overlapping read pairs (minimum 11-bp overlap). These analysis-ready reads were mapped against the *Phasianus colchicus* genome (Nuclear: GCA_004143745, Mitochondrial: NC_015526) using Bowtie2 [11]. The resulting alignment files were processed using Samtools [12] (coordinate sort, duplicate removal), followed by analysis of DNA fragmentation and terminal base damage using mapDamage (V2) [13].

Variant calling was performed on rescaled alignment files (output from mapDamage), using the 'mpileup' feature in Samtools. Only sites with >5x coverage were used for variant analysis. Consensus FASTA sequences, generated from variant files for each sample, were aligned with 34 complete mitochondrial genomes belonging to organisms in the *Phasianinae* sub-family, followed by construction of a Maximum likelihood tree using RAxML [14](GTRGAMMA substitution model, 100 bootstrap replicates).

Based on a SNP table (Supplemental Table 6) for the three mitogenomes across eight sites, specimens LM-092 and LM-093 are identical, while LM-091 differs from these at all eight sites. Therefore, LM-091 clearly represents a different individual and though LM-092 and LM-093 may come from different, but related individuals, we cannot rule out that these two specimens come from the same individual.

<u>Assessment of the Number of Individuals Analyzed in this Study</u>

Originally we selected bird bone from eight unique depositional contexts from the same archaeological site to analyze, with the expectation that these eight bones must have come from eight different individuals. However, because some of the molecular results are so similar we've had to take a closer look at the possibility that some of these bones in fact came from the same individual. Based on the similarities and differences of archaeological provenience, absolute age, skeletal element, diet, and mitogenome (Supplemental Table 7) the bird bone assemblage presented here represents a minimum of four individual birds but as many as eight. Even when the mitogenomes are identical, we cannot rule out the possibility that they come from different, perhaps very closely related, individuals

Supplemental Table 1. Description of the primers used in this study. All primers were designed in this study, except COI-F/R which were described by Xiang et al. [4]. Primer coordinates are relative to the indicated reference sequences. Note that the AmbigF1/R1 were designed to work for North American birds, but they work on some of the Dadiwan specimens are reported here relative to *Phasianus colchicus.*

Target Region	Primer	Reference Sequence	Coordinates to mtDNA	Sequence (5' to 3')	Amplicon Length	Species Targeted	Annealing Temperature
COI	COI-F	*Gallus gallus* NC_001323	7038-7056	CCTTTAGCCGGCAACCTAG	156	*Gallus gallus*	60°C
	COI-R		7173-7193	TAGGGGTGTTTGGTATTGTGA			
COI	AmbigF1	*Phasianus colchicus* NC_015526.1	6841-6862	TCCCMCGAATAAAYAACATAAG	175	North American birds	55°C
	AmbigR1		6994-7015	AGGTGDAGGGAGAAGATGGCYA			
COI	GALLUSF	*Gallus gallus* KX987152.1	7132-7152	GAGCCATCAACTTTATCACTA	167	*Gallus gallus*<br>*Gallus sonneratii*	58°C
	GALLUSR		7280-7298	AAGGTTGCGGTCGGTAAGT			
12S	SyrmF	*Syrmaticus reevesii* NC_010770.1	1492-1511	ATCAATAGCCACCCGGCGTA	196	*Syrmaticus ellioti*<br>*Syrmaticus humiae*<br>*Syrmaticus reevesii*	62°C
	SyrmR		1667-1687	ATCAAGATTTAGGGCCAGGCA			
COI	LophuraF	*Lophura nycthemera* JN709934.1	70-90	CACTTTTGGGAGATGACCAGA	189	*Lophura edwardsii*<br>*Lophura nycthemera*	58°C
	LophuraR		240-258	AAGAGGGGGGAAGAAGTCA			
COI	TetraF	*Tetraophasis obscurus* JF921876.1	6661-6678	AGCCGGCACTGCCCTAAG	198	*Tetraophasis obscurus*<br>*Tetraophasis szechenyii*	60°C
	TetraR		6840-6858	AATGCTATGTCTGGGGCGC			
COI	Pheas1F	*Crossoptilon mantchuricum* KY070317.1	6821-6839	CCAGACATAGCATTCCCAC	198	*Crossoptilon mantchuricum*<br>*Ithaginis cruentus*	58°C
	Pheas1R		7000-7018	TACACCTGCGAGATGAAGG			
COI	Pheas2F	*Phasianus colchicus* NC_015526.1	6723-6742	AATGTAATCGTCACAGCCCA	162	*Chrysolophus pictus*<br>*Phasianus colchicus*<br>*Pucrasia macrolopha*	58°C
	Pheas2R		6867-6884	GGAGGGAGGGAGAAGTCA			
COI	Pheas3F	*Tetraogallus altaicus* GQ482760.1	325-343	ATGAACTGTCTATCCACCC	195	*Tetraogallus altaicus*<br>*Tragopan temminckii*	56°C
	Pheas3R		501-519	ATGAGGACGGATCATACGA			
CYTB	PartF	*Alectoris magna* HQ727950.1	62-79	AAACGGCGCCTCATTCTT	204	*Alectoris chukar*<br>*Alectoris magna*<br>*Perdix dauurica*	54°C
	PartR		246-265	GCTGAGAATAGATTTGTGAT			
COI	OtisF	*Otis tarda* KY754526.1	63-81	GCGCTGAACTTGGCCAACC	186	*Otis tarda*<br>*Tetrax tetrax*	56°C
	OtisR		228-248	TGTTGTTTATGCGGGGGAATG			
COI	Pheas6841F	*Phasianus colchicus* NC_015526.1	6841-6862	TCCCACGTATAAACAACATAAG	175	*Phasianus colchicus*	62°C
	Pheas7015R		6994-7015	AGATGGAGGGAGAAAATGGCCA			

Supplemental Table 1. Continued

Target Region	Primer	Reference Sequence	Coordinates to mtDNA	Sequence (5' to 3')	Amplicon Length	Species Targeted	Annealing Temperature
Control Region	C149F	*Phasianus colchicus* NC_015526.1	149-169	ATGTAGACGGACATTACACCT	177	*Phasianus colchicus*	60°C
	C325R		306-325	GTCGAGCATAACCAAATGGG			
Control Region	C266F	*Phasianus colchicus* NC_015526.1	266-288	ATGAATGGTTACAGGACATACAT	173	*Phasianus colchicus*	62°C
	C438R		421-438	AGGGGCGAGGGGTGTAGG			
Control Region	C400F	*Phasianus colchicus* NC_015526.1	400-418	GCTTCAGGCCCATTCTTTC	181	*Phasianus colchicus*	58°C
	C580R		560-580	AACAGCAATGAAGAGAGAAGA			
Control Region	C539F	*Phasianus colchicus* NC_015526.1	539-556	CTCAAGTCCGTGATCGCG	173	*Phasianus colchicus*	58°C
	C711R		694-711	GTCTCATCGAGGGACACG			
Control Region	C660F	*Phasianus colchicus* NC_015526.1	660-678	ACTACTCCTGCGTTGCGTC	174	*Phasianus colchicus*	60°C
	C833R		812-833	GTAAAAATATGTCCGACAAGCA			
Control Region	C791F	*Phasianus colchicus* NC_015526.1	791-811	TAAATGGTGCTATTTAGTGAA	181	*Phasianus colchicus*	54°C
	C971R		953-971	TGGTTTGGGGGTTTTTATG			
Control Region	C931F	*Phasianus colchicus* NC_015526.1	931-952	ACTAAATTAACTAAAATTACCG	186	*Phasianus colchicus*	54°C
	C1116R		1093-1116	TATCATGAATATAAATAATGTTTA			
Control Region	C1068F	*Phasianus colchicus* NC_015526.1	1068-1088	TACTATAGGACAAAACCCCAC	182	*Phasianus colchicus*	56°C
	C1249R		1228-1249	CAAAAATCAATAGTAAGGTTAG			

Supplemental Table 2. Amplification results using the eight primer pairs that target short, overlapping section of the mitochondrial control region. Check marks (✓) indicate successful amplification, followed by the number of independent replicates that were sequenced. Sections that failed to amplify are indicated by "O" symbols. The asterisk (*) denotes that for the second amplicon produced, the reverse sequence failed. The daggers (†) denote that for the second amplicon produced, the forward sequence failed. The double dagger (‡) denote an amplicon for which the forward sequenced failed. As an example, using primers C149F/C325R (Supplemental Table 1), the sample LM-092 was sequenced four times. This same sample was sequenced twice using primers C791F/C971R, but for the second amplification, the forward sequence failed.

	Primers							
**Sample**	**C149F/C325R**	**C266F/C438R**	**C400F/C580R**	**C539F/C711R**	**C660F/C833R**	**C791F/C971R**	**C931F/C1116R**	**C1086F/C1294R**
**LM-089**	✓ (2)	O	O	O	✓ (1)	O	O	✓ (1)
**LM-090**	O	O	O	✓ (1)	O	✓ (2)	O	O
**LM-091**	✓ (3)	✓ (5)	✓ (2)	✓ (3)	✓ (1)	✓ (3)	O	✓ (1)
**LM-092**	✓ (4)	✓ (4)	✓ (2)	✓ (2)*	✓ (2)†	✓ (2)†	O	✓ (2)
**LM-093**	✓ (3)	✓ (2)	✓ (1)	✓ (2)	✓ (2)	✓ (1)	O	✓ (1)*
**LM-094**	✓ (2)	✓ (1)	✓ (1)	✓ (1)	✓ (1)	✓ (1)	O	✓ (1)
**LM-095**	✓ (2)	O	✓ (1)	O	O	O	O	✓ (1)

Supplemental Table 3. Mitochondrial DNA control region sequences. Sample sequences are reported relative to a *Phasianus colchicus* mitochondrial reference sequence (Genbank accession NC_015526.1). Question marks (?) indicate missing data and periods (.) indicate nucleotide states that do not differ from that of the reference sequence.

	Nucleotide Position														
Sample	170	171	194	196	233	251	290	296	304	683	790	913	1142	1210	Sequence read (nucleotide positions)
Phasianus colchicus	C	A	G	T	G	T	T	T	C	C	T	A	T	T	170-1228
**LM-089**	T	G	A	C	A	C	C	C	T	T	C	.	C	A	170-305, 679-811, 1090-1228
**LM-090**	?	?	?	?	?	?	?	?	?	T	?	T	?	?	557-693, 812-952
**LM-091**	T	G	A	C	A	C	C	C	.	T	C	.	C	A	170-952, 1090-1228
**LM-092**	T	G	A	C	A	C	C	C	T	T	C	T	C	A	170-952, 1090-1228
**LM-093**	T	G	A	C	A	C	C	C	T	T	C	T	C	?	170-952, 1090-1198
**LM-094**	T	G	A	C	A	C	C	C	T	T	C	.	C	A	170-952, 1090-1228
**LM-095**	T	G	A	C	A	C	C	C	.	?	?	T	C	A	170-305, 419-559, 1090-1228

Supplemental Table 4. Summary from shotgun DNA sequencing. Reads were quality filtered using AdapterRemoval. Analysis ready reads were compared to *P. colchicus* reference genome using Bowtie2.

Library	Raw Reads	Analysis Ready Reads	Percent Retained	Read mapping to *P. colchicus* whole genome				
				Percent Mapped	Percent Duplication	5’ C->T	3’ G->A	Median Fragment Size
LM-091	4,736,194	3,784,138	80%	79%	23%	5.8%	5.9%	55 bp
LM-092	6,459,625	5,859,974	91%	90%	16%	3.4%	2.8%	76 bp
LM-093	5,381,768	4,365,460	81%	87%	22%	3.7%	3.3%	74 bp
LM-094	2,166,186	955,670	44%	9%	38%	11.7%	11.6%	47 bp

Supplemental Table 5. Summary from mtDNA genome analysis.

Library	Analysis Ready Reads	Read mapping to *P. colchicus* mitochondrial genome			
		Reads Mapped	Unique Reads	Sites Covered (1X)	Sites Covered (5X)
LM-091	3,784,138	7,799	5,362	16,685	16,659
LM-092	5,859,974	9,171	6,714	16,692	16,679
LM-093	4,365,460	6,548	4,609	16,687	16,638
LM-094	955,670	315	222	9,812	127

Supplemental Table 6. SNP table for the three mitogenomes of three specimens (LM-091, LM-092, LM-093) across eight sites, specimens LM-092 and LM-093 are identical, while LM-091 differs from these at all eight sites.

Site:	303	912	4581	4839	9009	11670	14109	14464
Library								
LM-091	C	A	A	A	T	G	C	G
LM-092	T	T	G	G	C	A	T	A
LM-093	T	T	G	G	C	A	T	A

Supplemental Table 7. Matrix evaluation of similarities and differences of specimens, and whether they may come from the same individual animal.

	LM-088	LM-089	LM-090	LM-091	LM-092	LM-093	LM-094	LM-095	Possible Match
**LM-088**	-	proximate context; different element	distant context	distant context; different age	distant context; different age	distant context; different age	distant context; different age	distant context; different age	LM-089
**LM-089**	maybe different	-	distant context	distant context; different age	distant context; different age	distant context; different age	distant context; different age	distant context; different age	LM-088
**LM-090**	different	different	-	distant context; different age	distant context; different age	distant context; different age	distant context; different age	distant context; different age	no match
**LM-091**	different	different	different	-	distant context; different mitogenome	distant context; different mitogenome	distant context	distant context	no match
**LM-092**	different	different	different	different	-	proximate context; different diet; identical mitogenome	proximate context; similar diet; different element	proximate context; different diet; different element	LM-093 LM-094 LM-095
**LM-093**	different	different	different	different	possibly the same	-	proximate context; different diet; different element	proximate context; different diet; different element	LM-092 LM-094 LM-095
**LM-094**	different	different	different	different	possibly the same	maybe different	-	proximate context; different diet; different element	LM-092 LM-093 LM-095
**LM-095**	different	different	different	different	maybe different	maybe different	maybe different	-	LM-092 LM-093 LM-094
**Maximum # of Matches**	1	1	0	0	3	3	3	3	

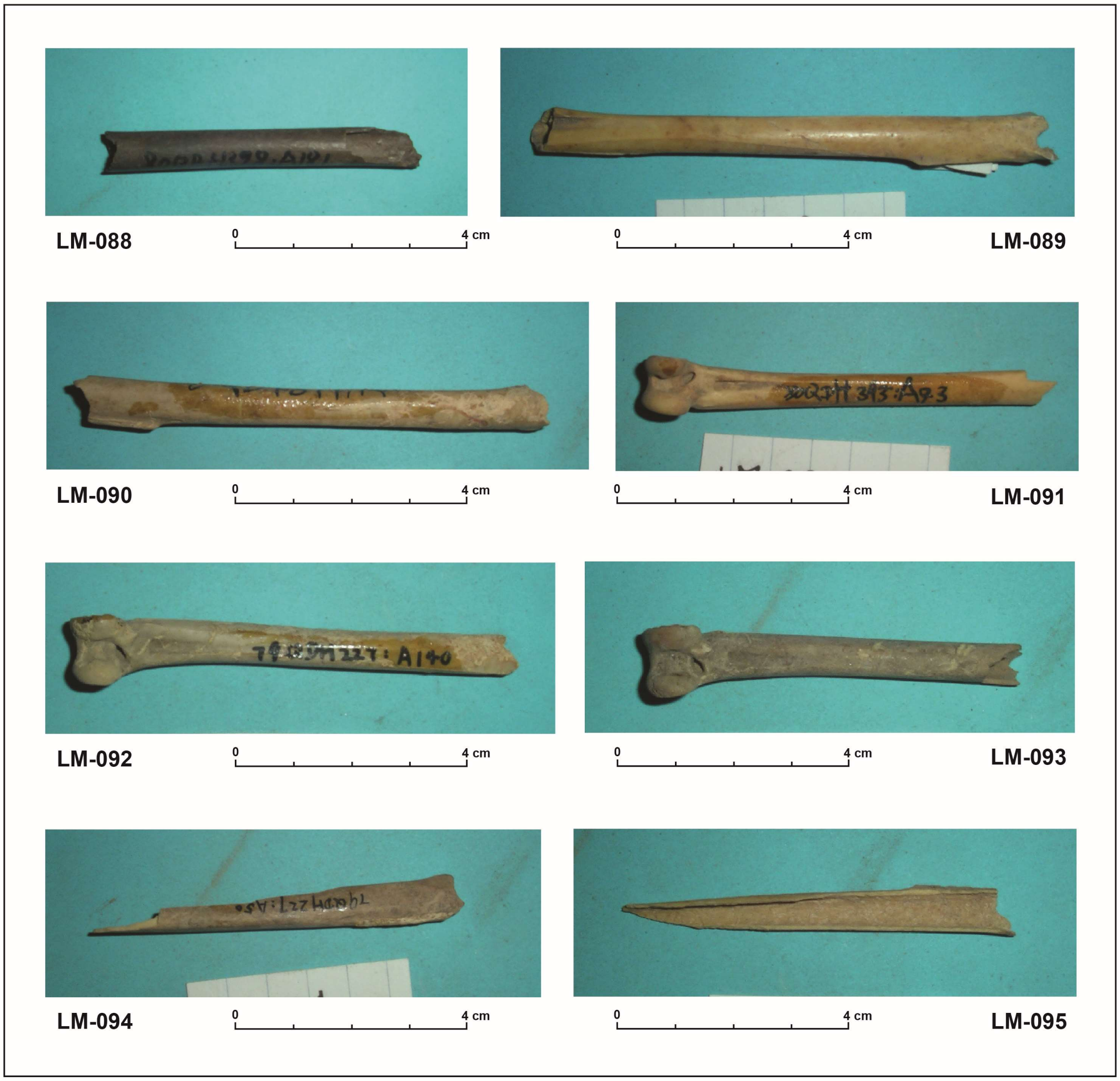

Supplemental Figure 1. Dadiwan bird remains analyzed in the current study. At the time of collection (March 2007) all specimens were curated at the Gansu Provincial Museum in Lanzhou, China. See [15-17] for original study.

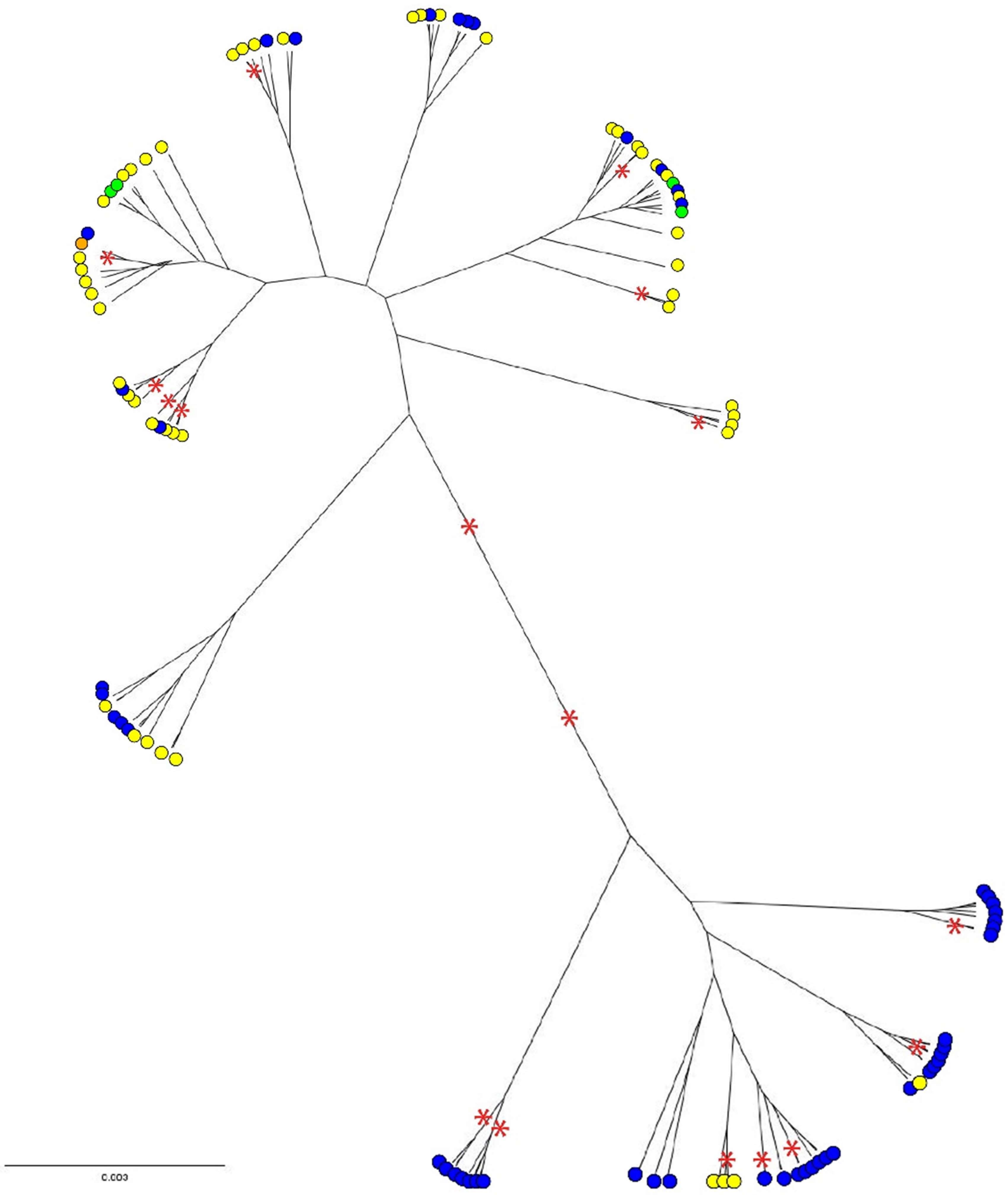

Supplemental Figure 2. Unrooted Bayesian tree illustrating the relationship between the Dadiwan specimens and 103 comparative control region sequences from Genbank. Green circles mark the Dadiwan specimens, blue circles represent comparative *P. c. pallasi* individuals, yellow circles comparative *P. c. strauchi* individuals, and the orange circle a comparative *P. c. alashanicus* individual. The red asterisks indicate branches with posterior probabilities greater than 0.50.

References

1. Kemp, B.M., et al., *Evaluation of methods that subdue the effects of polymerase chain reaction inhibitors in the study of ancient and degraded DNA.* Journal of Archaeological Science, 2014. **42**: p. 373-380.
2. Barta, J.L., C. Monroe, and B.M. Kemp, *Evaluation of the efficacy of contamination removal from bone surfaces.* Forensic Science International, 2013. **231**: p. 340-348.
3. Kemp, B.M., et al., *Prehistoric mitochondrial DNA of domesticate animals supports a 13th century exodus from the northern US southwest.* PLoS ONE, 2017. **12**: p. e0178882.
4. Xiang, H., et al., *Early Holocene chicken domestication in northern China.* Proceedings of the National Academy of Sciences, 2014. **111**(49): p. 17564-17569.
5. Kerr, K.C., et al., *Comprehensive DNA barcode coverage of North American birds.* Molecular Ecology Notes, 2007. **7**: p. 535-543.
6. Zhang, Z., M.B. Kermekchiev, and W.M. Barnes, *Direct DNA amplification from crude clinical samples using a PCR enhancer cocktail and novel mutants of Taq.* Journal of Molecular Diagnostics, 2010. **12**: p. 152-161.
7. Palmer, E., S. Tushingham, and B.M. Kemp, *Human use of small forage fish: improved ancient DNA species identification techniques reveal long term record of sustainable mass harvesting of smelt fisher in the northeast Pacific Rim.* Journal of Archaeological Science, 2018. **99**(143-152).
8. Carøe, C., et al., *Single- tube library preparation for degraded DNA.* Methods in Ecology and Evolution, 2017. **9**: p. 410-419.
9. Rohland, N., et al., *Partial uracil-DNA-glycosylase treatment for screening of ancient DNA.* Philosophical Transactions of the Royal Society B, 2015. **3370**: p. 20130624.
10. Schubert, M., S. Lindgreen, and L. Orlando, *AdapterRemoval v2: rapid adapter trimming, identification, and read merging.* BMC Research Notes, 2016. **9**: p. 88.
11. Langmead, B. and S.L. Salzberg, *Fast gapped-read alignment with Bowtie 2.* Nature Methods, 2012(9): p. 357-359.
12. Li, H., et al., *The Sequence Alignment/Map format and SAMtools.* Bioinformatics, 2009. **25**: p. 2078-2079.
13. Jonsson, H., et al., *mapDamage2.0: fast approximate Bayesian estimates of ancient DNA damage parameters.* Bioinformatics, 2013. **29**: p. 1682-1684.
14. Stamatakis, A., *RAxML version 8: a tool for phylogenetic analysis and post-analysis of large phylogenies.* Bioinformatics, 2014. **30**: p. 1312-1313.
15. Barton, L., et al., *Agricultural origins and the isotopic identity of domestication in northern China.* Proceedings of the National Academy of Sciences, 2009. **106**(14): p. 5523-5528.
16. Barton, L., *Early Food Production in China's Western Loess Plateau*, in *Anthropology*. 2009, PhD Dissertation, University of California, Davis: Davis, CA.

17. Barton, L., C. Morgan, and R.L. Bettinger, *Harvests for the hunters: the origins of food production in arid northern China.* The SAA Archaeological Record, 2009. **9**(3): p. 28-31.
